# A Novel Joint Power and Feedback Bit Allocation Interference Alignment Scheme for Wireless Sensor Networks

**DOI:** 10.3390/s17030563

**Published:** 2017-03-10

**Authors:** Shibao Li, Chang He, Yixin Wang, Yang Zhang, Jianhang Liu, Tingpei Huang

**Affiliations:** College of Computer and Communication Engineering, China University of Petroleum (East China), Qingdao 266580, China; chang.he@s.upc.edu.cn (C.H.); wangyixin.upc@gmail.com (Y.W.); zhangyang@upc.edu.cn (Y.Z.); liujianhang@upc.edu.cn (J.L.); huangtingpei@upc.edu.cn (T.H.)

**Keywords:** wireless sensor networks, MIMO, interference alignment, limited feedback, power allocation, throughput

## Abstract

It is necessary to improve the energy efficiency of batteries in wireless sensor networks (WSNs). The multiple-input multiple-output (MIMO) technique has become an important means to ameliorate WSNs, and interference management is the core of improving energy efficiency. A promising approach is interference alignment (IA), which effectively reduces the interference and improves the throughput of a system in the MIMO interference channels. However, the IA scheme requires perfect channel state information (CSI) at all transceivers in practice, which results in considerable feedback overhead. Thus, limited IA feedback has attracted much attention. In this paper, we analyze the throughput loss of the *K*-user MIMO interference channels when each transmitter delivers multiple streams in one slot, and derives the upper-bound of the system interference leakage and throughput loss. Then, to reduce the interference leakage and throughput loss for the MIMO interference alignment with limited feedback, a joint power and feedback bit allocation optimization scheme is proposed. The simulation results show that, compared with the conventional schemes, the presented optimal scheme achieves less residual interference and better performance in the system throughput.

## 1. Introduction

Composed of inexpensive and low-power-consumption sensors that have the ability to compute and communicate, wireless sensor networks (WSNs) have various applications in military defense, environmental testing, space exploration, home intelligence, traffic surveillance, and other fields [1]. However, it is hard to supply energy for battery-powered sensors in bad environments. Thus, improving the efficiency of energy utilization is becoming a challenge for WSNs researchers. As a key technology to promote the energy efficiency in modern wireless communications, the multiple-input multiple-output (MIMO) technique can improve the throughput of a system without increasing the transmitted power. WSNs have lower transmitted power and longer service lives by using the MIMO technique; thus, MIMO has become an important means of ameliorating the WSNs [2,3].

As the scale and quantity of networks are increasing, the significant advantages of MIMO are limited by the interference of multiple users sharing the same channel, especially when the channel conditions are poor. Interference alignment (IA) is a promising approach, that effectively reduces the interference in MIMO interference channels. By designing precoding and decoding matrices, IA allocates higher dimensional subspace for desired receivers, and aligns the interference from different sources in the receivers [4]. By using the IA concept, a system can achieve the maximum degrees of freedom (DoF) [5]. In the *K*-user interference channels, each user can achieve 1/2 DoF, and guarantee the total K/2 DoF, although the number of users increases [6]. Encouraged by this surprising result, much work has been done to apply IA to different interference networks, such as MIMO networks [7], cellular networks [8,9,10], cognitive radio [11], ad hoc networks [12], etc.

For most IA approaches, the global and perfect channel state information (CSIT) must be available at each transmitter [13,14,15,16]. However, in practical situations such as in frequency division duplex (FDD) systems, the condition is difficult to implement. In FDD systems, the CSI needs to be obtained from the feedback channel. In traditional MIMO systems, limited feedback based on the quantization codebook is a common and powerful method to help the transmitters obtain the CSI from the receivers [17]. Similarly, with the IA over MIMO interference channels, the limited feedback scheme is also discussed in [18,19]. In [18], for a frequency selective single-input single-output (SISO) channel with *L* taps in the channel between any pair of nodes, and as the total power *P* available with the transmitting sources, it is shown that by analyzing the limited feedback scenario, the full spatial multiplexing gain can still be obtained as long as the feedback rate is scaled as K(L−1)logP bits per receiver. In [19], the work is extended to the MIMO interference channel, and the Grassmannian manifold based on the limited feedback technique introduced into the MIMO interference channels, where the relationship between the performance of IA and the feedback amount or codebook size is revealed. It is found that even with limited CSI feedback, the maximum degrees of freedom for the interference channel can be achieved. It is well known that the Grassmannian codebooks are optimal for independent identically distributed channels [18,19], but it is challenging to design the optimal codebooks except in some special cases. Therefore, it is impractical to use the Grassmannian codebooks for limited feedback. Random vector quantization (RVQ) codebooks perform close to the Grassmannian codebooks for wireless channels with independent identically distributed gain [20,21]. An IA scheme with RVQ-based quantized transmitting precoders for the MIMO interference channels is proposed in [22]. The authors in [23] make use of the limited feedback theory to analyze the performance of the subspace IA in uplink cellular systems. Furthermore, the subspace IA scheme with limited feedback is optimized by minimizing the chordal distance of real CSI and Grassmannian quantization codeword in [24], and the outage capacity is analyzed for the MIMO interference channels employing IA with limited feedback in [25].

For IA based on limited CSI feedback, the throughput of an IA interference network is reduced by the residual interference (due to imperfect IA) compared with perfect CSI [26,27]. To minimize the performance loss, it is necessary to take some effective performance optimization measures. An upper bound on rate loss caused by limited feedback is derived, and a beamformer design method is given to minimize the upper bound in [28]. In [29], an improved metric to measure the performance of IA under limited CSI feedback is presented, which can better reflect the throughput degradation due to limited feedback. Considering that the total feedback amount is constrained in a practical system (due to limited feedback capacity), in order to improve the system performance, we should distribute the feedback resource among the forward and interference channels according to channel conditions. Therefore, [30] presents a feedback allocation scheme for IA in the limited-feedback MIMO interference channel with a single data stream for each link by minimizing the average residual interference. Then, the feedback allocation scheme is extended to a case with multiple data streams [31], considering heterogeneous path loss and spatial correlations, and a dynamic quantization scheme via bit allocations is proposed.

Another consideration is that power allocation plays an important role in improving the energy efficiency and throughput in interference networks [32,33]. In [32], a cooperative Nash bargaining resource allocation algorithm based on power control and sub-channel scheduling gives a good trade-off between throughput and fairness. In [33], an iterative sub-channel and power allocation algorithm is proposed to maximize the total capacity. In theory, the total throughput can be promoted by combining the allocation of transmitted power and feedback bits. However, there is a complicated relationship between them, as they influence and restrict each other in practice. Therefore, it is urgent to solve the problem of how to analyze the impact of mutual relationships on the performance of a system.

Among the existing literature, there has been no thorough investigation of the relationship between transmitted power and feedback bits or the transmitted power of multiple data streams equally allocated in the IA interference network. Therefore, this paper formulates the transmitted power and feedback bit allocation problem in the *K*-user MIMO interference channels, and a joint power and feedback-bit allocation scheme (JPFAS) is proposed to reduce the residual interference and improve the throughput of the IA interference network.

The rest of this paper is organized as follows. Section 2 introduces the system model. In Section 3, the CSI quantization and system throughput loss are analyzed. In Section 4, the JPFAS is proposed. Simulation results to evaluate the proposed algorithm are presented and discussed in Section 5, followed by the conclusion and future work in Section 6.

Notations: the bold upper (lower) letters denote matrices (column vectors), and ${\left(\cdot \right)}^{T}$ and ${\left(\cdot \right)}^{H}$ denote transposition and conjugate transpose. E[⋅] denotes expectation, |⋅| denotes the absolute value, ||⋅|| and $||\cdot |{|}_{F}$ denote the L2-norm, and Frobenius norm respectively. vec(A) denotes matrix vectorization.

## 2. System Model

**Figure 1:**
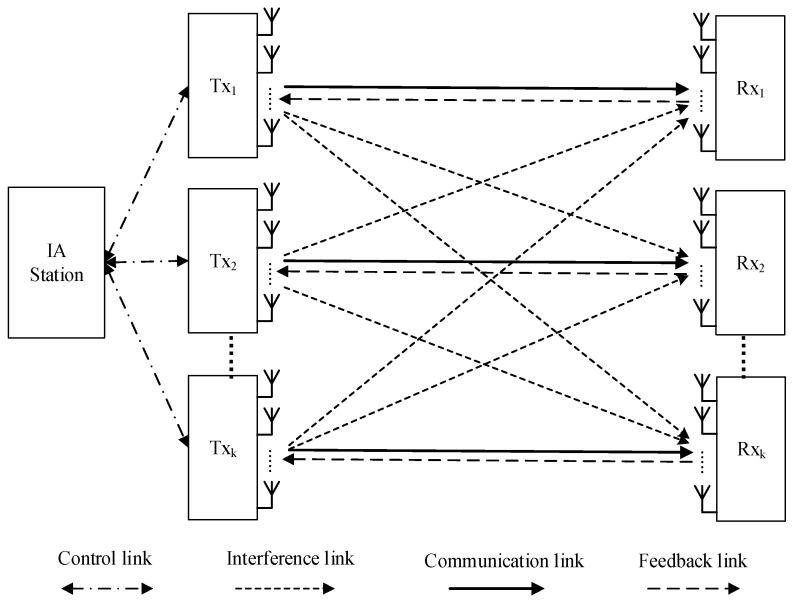
*K-*user multiple-input multiple-output (MIMO) limited feedback interference channel model.

As is shown in Figure 1, we consider the *K*-user MIMO interference channels with limited feedback where transmitters and their desired receivers are equipped with *N* antennas respectively. *D* data streams are transmitted by each transmitter, and *D* is limited by D≤N to ensure the implementation of IA. We assume that there is an IA station controlling the reconstruction of quantized CSI collected from all transmitters, the transmitting power of each stream, and the dynamic allocation for feedback bits. All statistics of the fading channels characterized by large-scale fading are independent and identically distributed (i.i.d.). In the same slot, the antennas’ characteristic remains unchanged. The *d*-th data stream at the *k-*th receiver is given by

(1) $$\begin{array}{l} y_{k}={\left(u_{k}^{d}\right)}^{H}\sqrt{\eta_{k,k}p_{k,d}}H_{k,k}v_{k}^{d}s_{k}^{d}\\ \;+{\left(u_{k}^{d}\right)}^{H}\sum_{j=1,j\neq d}^{D}\sqrt{\eta_{k,k}p_{k,j}}H_{k,k}v_{k}^{j}s_{k}^{j}\\ \;+{\left(u_{k}^{d}\right)}^{H}\sum_{i=1,i\neq k}^{K}\sum_{j=1}^{D}\sqrt{\eta_{k,i}p_{k,j}}H_{k,i}v_{i}^{j}s_{i}^{j}+{\left(u_{k}^{d}\right)}^{H}n_{k} \end{array}$$

The first item is the desired signal, the middle items are intra-user and inter-user interference, and the last denotes the Gaussian noise with zero mean. $s_{k}={\left[s_{k}^{1},\cdots ,s_{k}^{D}\right]}^{T}$ means the *D* i.i.d. streams transmitted from the same transmitter. $V_{i}=[v_{i}^{1},\cdots ,v_{i}^{D}]$ denotes precoding matrix of transmitter *i*, and $U_{i}=[u_{i}^{1},\cdots ,u_{i}^{D}]$ is corresponding receiving filter matrix. The elements in $V_{i}$ and $U_{i}$ meet the condition that $||v_{i}^{j}{\left|\right|}_{F}=||u_{i}^{j}{\left|\right|}_{F}=1$. Channel matrix $H_{k,i}$ comprises i.i.d. elements distributed complex Gaussian random variables with zero mean and unit variance. $n_{k}$ is Gaussian noise with $n_{k}∼CN(0,\sigma^{2}I_{N})$. $p_{k,j}$ is the transmitting power that transmitter *k* allocates to the *j-*th data stream, $\eta_{k,i}$ means the path loss from transmitter *i* to receiver *k*. According to the channel model raised by ITU-R (Radiocommunication Sector of the International Telecommunication Union), $\eta_{k,i}$ is given by

(2) $$\eta_{k,i}=40\mathrm{lg}r_{k,i}+30\mathrm{lg}f+49[dB]$$

where $r_{k,i}$ is the distance between transmitter *i* and receiver *k*, *f* is the frequency of the carrier signal that takes the fixed value at 2 GHz.

Supposing that perfect CSI is available at all transmitters, the interference needs to be aligned into a uniform subspace for the achievement of IA which demands the system to satisfy

(3) $$\{\begin{array}{c} {\left(u_{k}^{d}\right)}^{H}H_{k,i}v_{i}^{j}=0,\forall k\neq i\\ {\left(u_{k}^{d}\right)}^{H}H_{k,k}v_{k}^{j}=0,\forall d\neq j\\ \left|{\left(u_{k}^{d}\right)}^{H}H_{k,k}v_{k}^{d}\right|>0,\forall k,d \end{array}$$

The throughput of system with perfect CSI is

(4) $$\begin{array}{cc} R_{\sum } & =\sum_{k=1}^{K}\sum_{d=1}^{D}R_{k}^{d}\\ & =\sum_{k=1}^{K}\sum_{d=1}^{D}\log_{2}\left(1+\frac{p_{k,d}\eta_{k,k}{\left|{\left(u_{k}^{d}\right)}^{H}H_{k,k}v_{k}^{d}\right|}^{2}}{\sigma^{2}}\right) \end{array}$$

## 3. CSI Quantization and Throughput Loss Analysis

**Figure 2:**
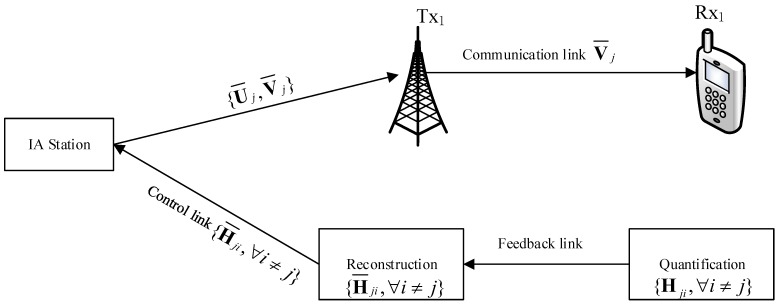
Limited feedback topology for MIMO interference network.

To reduce the considerable overheads caused by CSI feedback, receivers get the perfect CSI from channel estimation without computing estimation error, and feedback the perfect CSI quantization to their corresponding transmitters through feedback links as shown in Figure 2.

As the optimal codebook for quantizing the MIMO channels, the Grassmannian codebook has been widely used in CSI quantization. However, designing the codebook and computing its high-precision quantization is highly complex. Compared with the conventional Grassmannian quantization, RVQ is superior in terms of its realistic feedback quantization progress because of its easier design for codebook, lower computational complexity and approximate quantization performance. Therefore, RVQ is chosen to quantize perfect CSI in this paper. The channel matrix $H_{k,i}$ is expanded into a vector $h_{k,i}$. Then based on the local codebook, $h_{k,i}$ is quantized at receiver as

(5) $${\bar{h}}_{k,i}=\arg\underset{\omega_{m}\in \mathbb{C}}{\min}\mathrm{dist}\left(h_{k,i},w_{m}\right)$$

where $w_{m}$ is of unit-norm, *B* denotes the number of feedback bits. The size of codebook is $2^{B}$. The chordal distance between vector $h_{k,i}$ and codeword $w_{m}$ is defined by $dist(h_{k,i},w_{m})=\sqrt{1-|h_{k,i}^{H}w_{m}{|}^{2}}$. The labels corresponding to the selected codewords are transmitted to transmitter *k*. After reconstructing CSI by finding out codewords from the same codebook according to the labels, transmitters obtain the quantized CSI $\left\{{\bar{H}}_{k,i}\right\}$. Finally, transmitters use the feedback CSI to calculate the precoding matrices and receiving filter matrices to achieve interference alignment that requires the following conditions to be met

(6) $$\{\begin{array}{c} {\left({\bar{u}}_{k}^{d}\right)}^{H}{\bar{H}}_{k,i}{\bar{v}}_{i}^{j}=0,\forall k\neq i\\ {\left({\bar{u}}_{k}^{d}\right)}^{H}{\bar{H}}_{k,k}{\bar{v}}_{k}^{j}=0,\forall d\neq j\\ \left|{\left({\bar{u}}_{k}^{d}\right)}^{H}{\bar{H}}_{k,k}{\bar{v}}_{k}^{d}\right|>0,\forall k,d \end{array}$$

The throughput of system adopting IA with limited feedback is

(7) $$\begin{array}{cc} {\bar{R}}_{\sum } & =\sum_{k=1}^{K}\sum_{d=1}^{D}{\bar{R}}_{k}^{d}\\ & =\sum_{k=1}^{K}\sum_{d=1}^{D}\log_{2}\left(1+\frac{p_{k,d}\eta_{k,k}{\left|{\left({\bar{u}}_{k}^{d}\right)}^{H}H_{k,k}{\bar{v}}_{k}^{d}\right|}^{2}}{\sigma^{2}+{\bar{I}}_{k,1}^{d}+{\bar{I}}_{k,2}^{d}}\right) \end{array}$$

where ${\bar{I}}_{k,1}^{d}$ denotes the power of inter-user interference, ${\bar{I}}_{k,2}^{d}$ denotes the power of intra-user interference. ${\bar{I}}_{k,1}^{d}$ and ${\bar{I}}_{k,2}^{d}$ can be respectively described as

(8) $${\bar{I}}_{k,1}^{d}={\sum_{j=1,j\neq d}^{D}p_{k,j}\eta_{k,k}\left|{\left({\bar{u}}_{k}^{d}\right)}^{H}H_{k,k}\bar{v}{}_{k}^{j}\right|}^{2}$$

(9) $${\bar{I}}_{k,2}^{d}=\sum_{i=1,i\neq k}^{K}{\sum_{j=1}^{D}p_{i,j}\eta_{k,i}\left|{\left({\bar{u}}_{k}^{d}\right)}^{H}H_{k,i}\bar{v}{}_{i}^{j}\right|}^{2}$$

Perfect CSI $\left\{H_{k,i}\right\}$ can be expressed by quantized CSI $\left\{{\bar{H}}_{k,i}\right\}$ as

(10) $$H_{k,i}=\cos\theta_{k,i}{\bar{H}}_{k,i}+\sin\theta_{k,i}\Delta H_{k,i}$$

where $\theta_{i}=\arccos|H_{k,i}^{H}{\bar{H}}_{k,i}|$, $\Delta H_{k,i}$ is the vector of quantization error and $||\Delta H_{k,i}{\left|\right|}_{F}=||{\bar{H}}_{k,i}{\left|\right|}_{F}$. On the basis of Equation (10), we can analyze the influence of quantized CSI on the sum rate of the system.

Due to the estimation error of quantized CSI, the interference could not be absolutely eliminated. Some interference leaks to the desired subspace and results in the decrease of system throughput. The throughput loss for the *d-*th data stream at *k-*th receiver is recorded as $\Delta R_{k}^{d}=E\left[R_{k}^{d}-{\bar{R}}_{k}^{d}\right]$. Its upper-bound is derived by Jensen’s inequality as

(11) $$\begin{array}{cc} \Delta R_{k}^{d} & =E\left[\log_{2}\left(1+\frac{p_{k,d}\eta_{k,k}{\left|{\left({\bar{u}}_{k}^{d}\right)}^{H}H_{k,k}{\bar{v}}_{k}^{d}\right|}^{2}}{\sigma^{2}}\right)\right]-E\left[\log_{2}\left(1+\frac{p_{k,d}\eta_{k,k}{\left|{\left({\bar{u}}_{k}^{d}\right)}^{H}H_{k,k}{\bar{v}}_{k}^{d}\right|}^{2}}{\sigma^{2}+{\bar{I}}_{k,1}^{d}+{\bar{I}}_{k,2}^{d}}\right)\right]\\ & \leq E\left[\log_{2}\left(1+\frac{p_{k,d}\eta_{k,k}{\left|{\left({\bar{u}}_{k}^{d}\right)}^{H}H_{k,k}{\bar{v}}_{k}^{d}\right|}^{2}}{\sigma^{2}}\right)\right]+E\left[\log_{2}\left(1+\frac{{\bar{I}}_{k,1}^{d}}{\sigma^{2}}+\frac{{\bar{I}}_{k,2}^{d}}{\sigma^{2}}\right)\right]\\ & \;-E\left[\log_{2}\left(1+\frac{p_{k,d}\eta_{k,k}{\left|{\left({\bar{u}}_{k}^{d}\right)}^{H}H_{k,k}{\bar{v}}_{k}^{d}\right|}^{2}}{\sigma^{2}}+\frac{{\bar{I}}_{k,1}^{d}}{\sigma^{2}}+\frac{{\bar{I}}_{k,2}^{d}}{\sigma^{2}}\right)\right]\\ & \leq \log_{2}\left(1+E\left[\frac{{\bar{I}}_{k,1}^{d}}{\sigma^{2}}\right]+E\left[\frac{{\bar{I}}_{k,2}^{d}}{\sigma^{2}}\right]\right) \end{array}$$

The upper-bound shows that the rate loss logarithmically increases with the addition of residual interference. To minimize the rate loss created by quantizing CSI, the residual interference should be reduced as much as possible.

According to Equations (10) and (11), the residual interference of the intra-user at *k-*th receiver is

(12) $$\begin{array}{cc} {\bar{I}}_{k,2}^{d} & =\sum_{i=1,i\neq k}^{K}\sum_{j=1}^{D}(\eta_{k,i}p_{i,j}|{\left({\bar{u}}_{k}^{d}\right)}^{H}\left(\left(\cos\theta_{k,i}\right){\bar{H}}_{k,i}\right){+\left(\sin\theta_{k,i}\Delta H_{k,i}\right)){\bar{v}}_{i}^{j}|}^{2})\\ & =\;\sum_{i=1,i\neq k}^{K}\sum_{j=1}^{D}\left(\eta_{k,i}p_{i,j}\sin^{2}\theta_{k,i}{\left|{\left({\bar{u}}_{k}^{d}\right)}^{H}\Delta H_{k,i}{\bar{v}}_{i}^{j}\right|}^{2}\right)\\ & =\;\sum_{i=1,i\neq k}^{K}\sum_{j=1}^{D}(\eta_{k,i}p_{i,j}\sin^{2}\theta_{k,i}|vec{\left(\Delta H_{k,i}\right)}^{H}\;{\times vec\left(u_{k}^{d}{\left(v_{i}^{j}\right)}^{H}\right)|}^{2}) \end{array}$$

where $e_{k,i}^{d,j}=vec(u_{k}^{d}{\left(v_{i}^{j}\right)}^{H})$ and

(13) $${\left\|e_{k,i}^{d,j}\right\|}_{F}^{2}=\mathrm{tr}\left(u_{k}^{d}{\left(u_{k}^{d}\right)}^{H}\right)={\left\|u_{k}^{d}\right\|}_{F}^{2}=1$$

Thus, the expectation of intra-user residual interference at receiver *k* can be written as

(14) $$E\left[{\bar{I}}_{k,2}^{d}\right]\leq \sum_{i=1,i\neq k}^{K}\sum_{j=1}^{D}\eta_{k,i}p_{k,j}E\left[\sin^{2}\theta_{k,i}\right]\times E\left[{\left|vec{\left(\Delta H_{k,i}\right)}^{H}e_{k,i}^{d,j}\right|}^{2}\right]$$

The random variables $\sin^{2}\theta_{k,i}$ and $|vec{\left(\Delta H_{k,i}\right)}^{H}e_{k,i}^{d,j}{|}^{2}$ are independent. $|vec{\left(\Delta H_{k,i}\right)}^{H}e_{k,i}^{d,j}{|}^{2}$ is beta-distributed with an expectation that equals $1/(N^{2}-1)$. The expectation of $\sin^{2}\theta_{k,i}$ is given by $\bar{\Gamma }(N^{2})2^{-B_{k,i}/(N^{2}-1)}$ from [10] where $\bar{\Gamma }(N^{2})=\Gamma (1/(N^{2}-1))/(N^{2}-1)$. Assuming $B_{k,i}$ is the number of feedback bits from transmitter *i* to receiver *j*, we can rewrite Equation (14) as

(15) $$E\left[{\bar{I}}_{k,2}^{d}\right]\leq \sum_{i=1,i\neq k}^{K}\sum_{j=1}^{D}\eta_{k,i}p_{k,j}\frac{\bar{\Gamma }(N^{2})}{N^{2}-1}2^{-\frac{B_{k,i}}{N^{2}-1}}$$

Similarly, the expectation of inter-user can be obtained as

(16) $$E\left[{\bar{I}}_{k,1}^{d}\right]\leq \sum_{j=1,j\neq d}^{D}\eta_{k,i}p_{k,j}\frac{\bar{\Gamma }(N^{2})}{N^{2}-1}2^{-\frac{B_{k,i}}{N^{2}-1}}$$

Therefore, the expectation of total residual interference to noise ratio for the *d-*th stream at *k-*th receiver can be written as

(17) $$\begin{array}{cc} E\left[\frac{{\bar{I}}_{k}^{d}}{\sigma^{2}}\right] & =E\left[\frac{{\bar{I}}_{k,1}^{d}}{\sigma^{2}}\right]+E\left[\frac{{\bar{I}}_{k,2}^{d}}{\sigma^{2}}\right]\\ & \leq \sum_{i=1}^{K}\sum_{j=1}^{D}\frac{1}{\sigma^{2}}p_{k,j}a_{i,j}^{k}2^{-\frac{B_{k,i}}{N^{2}-1}} \end{array}$$

where $a_{i,j}^{k}$ is defined as

(18) $$a_{i,j}^{k}=\left\{ \begin{array}{l} \eta_{k,i}\frac{\bar{\Gamma }(N^{2})}{N^{2}-1},\;i\neq k\;\mathrm{or}\;j\neq d\\ 0,\;i=k\;,j=d \end{array}\right.$$

Combining Equation (11), we have

(19) $$\Delta R_{k}^{d}\leq \log_{2}\left(1+\sum_{i=1}^{K}\sum_{j=1}^{D}\frac{1}{\sigma^{2}}p_{k,j}a_{i,j}^{k}2^{-\frac{B_{k,i}}{N^{2}-1}}\right)$$

The sum rate loss of the *k-*th receiver is given by

(20) $$\Delta R_{k}=\sum_{d=1}^{D}\Delta R_{k}^{d}\leq D\log_{2}\left(1+\sum_{i=1}^{K}\sum_{j=1}^{D}\left(a_{i,j}^{k}\frac{p_{k,j}}{\sigma^{2}}2^{-\frac{B_{k,i}}{N^{2}-1}}\right)\right)$$

Thus, the actual rate achieved in receiver *k* is

(21) $$\begin{array}{cc} {\bar{R}}_{k} & =R_{k}-\Delta R_{k}\\ & \geq \sum_{d=1}^{D}\log_{2}\left(1+\frac{p_{k,d}\eta_{k,k}{\left|{\left(u_{k}^{d}\right)}^{H}H_{k,k}v_{k}^{d}\right|}^{2}}{\sigma^{2}}\right)-D\log_{2}\left(1+\sum_{i=1}^{K}\sum_{j=1}^{D}\left(a_{i,j}^{k}\frac{p_{k,j}}{\sigma^{2}}2^{-\frac{B_{k,i}}{N^{2}-1}}\right)\right) \end{array}$$

As shown in Equation (21), due to the interference leakage in the limited feedback networks, the low-bound of the throughput of a system can be determined by the difference between the throughput under perfect feedback networks and the loss of throughput caused by quantized errors. Through further analysis, it can be found that the throughput under perfect feedback network is a function of the allocation of transmitted power, and the loss of throughput is a function of power allocation and feedback bits allocation. Therefore, it is sufficient to optimize the distribution of transmitted power and feedback bits.

## 4. Joint Power and Feedback Bit Allocation Interference Alignment Scheme

As shown in the previous section, although IA can effectively mitigate the interference over the MIMO interference channel and improve the performance, the performance can be significantly degraded by the residual interference due to the limited feedback. Power allocation has been widely used to maximize the capacity of the multi-user network, and dynamic feedback bits allocation has been used to reduce the throughput loss due to limited feedback. However, researchers have not yet attempted to take the transmitted power and feedback bits into account synthetically to optimize the system performance. Thus, in this section, we first formulate the joint power and feedback bits allocation problem, aiming to maximize the throughput. Then, we discuss the relationship between the transmitted power and feedback bits in this problem. After decomposing the allocation problem into following sub-problems, we solve these problems by the Lagrangian methods and water-filling algorithm.

A. Problem Formulation and Analysis.

First, we formulate the joint power and feedback bits allocation problem for the *K-*user MIMO interference channels. The maximization of the sum rate loss of the *k-*th receiver with interference alignment can be mathematically formulated as the following problem:

(22) $$\begin{array}{cc} \max & \sum_{d=1}^{D}\log_{2}\left(1+\frac{p_{k,d}\eta_{k,k}{\left|{\left(u_{k}^{d}\right)}^{H}H_{k,k}v_{k}^{d}\right|}^{2}}{\sigma^{2}}\right)-D\log_{2}\left(1+\sum_{i=1}^{K}\sum_{j=1}^{D}\left(a_{i,j}^{k}\frac{p_{k,j}}{\sigma^{2}}2^{-\frac{B_{k,i}}{N^{2}-1}}\right)\right)\\ s.t. & \sum_{d=1}^{D}p_{k,d}\leq P_{k}\\ & \left\|{\bar{u}}_{k}^{d}\right\|=1,1<k<K,1<d<D\\ & \left\|{\bar{v}}_{k}^{d}\right\|=1,1<k<K,1<d<D\\ & \sum_{i=1}^{K}B_{k,i}<B_{f} \end{array}$$

It can be seen that the problem defined in Equation (22) is a nonconvex optimization problem. Although it can be solved by an intelligent algorithm or numerical search algorithm, these methods need to carry out considerable iterative operations leading to high computational complexity and long computational times, which are not conducive to practical application.

The aim of the interference alignment is to reduce the intensity of the interference signal, and power allocation mainly improves the transmission efficiency of the desired channel by optimizing the transmitted power of different sub-data streams. Considering the fact that two approaches are relatively independent, the power allocation and feedback bits allocation problem can be individually optimized to achieve the suboptimal system performance with low computational complexity. Based on the throughput loss analysis in the previous section, it is obvious that power allocation mainly depends on the strength of the desired channel matrix at moderate to high SNR. Therefore, we can allocate the power only according to the matrix of desired channel at first, and then design the feedback bits allocation scheme according to the allocated power and the large-scale fading of the interference channels. Thus, we decompose the problem in Equation (22) into the following sub-problems.

B. Power Allocation for the Transmitters.

(23) $$\begin{array}{l} \max\sum_{d=1}^{D}\log_{2}\left(1+\frac{p_{k,d}\eta_{k,k}{\left|{\left({\bar{u}}_{k}^{d}\right)}^{H}H_{k,k}{\bar{v}}_{k}^{d}\right|}^{2}}{\sigma^{2}}\right)\\ s.t.\;\sum_{d=1}^{D}p_{k,d}\leq P_{k} \end{array}$$

In order to solve the problem B, the equivalent channel matrix of transmitter *k* to receiver *k* is ${\overset{⌣}{H}}_{k,k}={\left({\bar{u}}_{k}^{d}\right)}^{H}H_{k,k}{\bar{v}}_{k}^{d}$. ${\overset{⌣}{H}}_{k,k}$ can be expressed based on singular value decomposition (SVD) in eigenvalue descending order as

(24) $${\overset{⌣}{H}}_{k,k}=ZS_{k,k}W^{H}$$

where Z and W are unitary matrices, singular values are arranged as the diagonal elements of the diagonal matrix $S_{k,k}$ in descending order. The optimization problem of the *k*-th receiver’s sum rate can be written as

(25) $$\begin{array}{l} \max\sum_{d=1}^{D}\log_{2}\left(1+\frac{s_{k,k,d}^{2}p_{k,d}\eta_{k,k}}{\sigma^{2}}\right)\\ s.t.\;\sum_{d=1}^{D}p_{k,d}\leq P_{k} \end{array}$$

where $s_{k,k,d}$ is the *d*-th element of the diagonal of the matrix $S_{k,k}$, $P_{k}$ is the total transmitted power of the transmitter *k*. Problem Equation (25) can be solved by the classic water-filling algorithm, and the transmitted power of each data stream can be found by

(26) $$p_{k,d}=\max\left(\lambda -\frac{\sigma^{2}}{s_{k,k,d}^{2}\eta_{k,k}},0\right)$$

where λ can be obtained by solving $\sum_{d=1}^{D}p_{k,d}=P_{k}$.

C. Feedback Bits Allocation for the Receivers.

To dynamically allocate the number of channel feedback bits to receiver *k*, the dynamic bit allocation problem can be described as

(27) $$\begin{array}{l} \min\sum_{i=1}^{K}\sum_{j=1}^{D}\left(a_{i,j}^{k}p_{k,j}2^{-\;\frac{B_{k,i}}{N^{2}-1}}\right)\\ s.t.\sum_{i=1}^{K}B_{k,i}\leq B_{f} \end{array}$$

where $B_{f}$ denotes the number of sum feedback bits, and $B_{k,i}$ is a non-negative integer.

Combining Equations (21) and (24), we dynamically allocate feedback bits based on channel loss. To solve the problem of Equation (21), we formulate the Lagrangian with multiplier γ. Then, we get

(28) $$L=\sum_{i=1}^{K}\sum_{j=1}^{D}\left(a_{i,j}^{k}p_{k,j}2^{-\frac{B_{k,i}}{N^{2}-1}}\right)+\gamma \sum_{i=1}^{K}\left(B_{k,i}-B_{f}\right)$$

Take the derivative with $B_{k,i}$ and γ respectively. We have

(29) $$\frac{\partial L}{\partial B_{k,i}}=-\ln2\frac{a_{i,j}^{k}p_{k,j}}{N^{2}-1}2^{\frac{-B_{k,i}}{N^{2}-1}}+\gamma =0$$

(30) $$\frac{\partial L}{\partial \gamma }=\sum_{i=1}^{K}B_{k,i}-B_{f}=0$$

Defining $\frac{\ln L}{r}=\mu$, $B_{k,i}$ is given by

(31) $$B_{k,i}=\left(N^{2}-1\right)\log_{2}\left(\frac{\mu a_{i,j}^{k}p_{k,j}}{N^{2}-1}\right)$$

Considering Equations (25) and (29), we have

(32) $$B_{f}=\sum_{k=1}^{K}\left(N^{2}-1\right)\log_{2}\left(\frac{\mu a_{i,j}^{k}p_{k,j}}{N^{2}-1}\right)$$

Applying the water-filling algorithm, the number of optimal feedback bit is

(33) $$B_{k,i}^{*}=\frac{1}{K}\left(r-\left(N^{2}-1\right)K\log_{2}\left(\frac{N^{2}-1}{a_{i,j}^{k}p_{k,j}}\right)\right)$$

where $r=B_{f}+\sum_{k=1}^{K}\left(N^{2}-1\right)\log_{2}\left(\frac{N^{2}-1}{a_{i,j}^{k}p_{k,j}}\right)$, $B_{k,i}^{*}$ is the smallest integer among those larger than $B_{k,i}^{*}$.

After considering the relative independence between the interference space and the space of the desired signal, the transmitted power and feedback bits are optimized individually to maximize the throughput by the allocation algorithm proposed in problem B and C. Then, we get precoding matrices and receiving filter matrices by the MinIL algorithm in [13].

## 5. Numerical Results

In this section, the effectiveness of the proposed JPFAS under the MIMO interference networks with limited feedback through simulation was verified. A multi-cell pattern containing three hexagons, where a transmitter is located in the center of the cell, and a receiver stands on the edge was considered. The distance between the transmitter and its corresponding receiver randomly distributes at the range of 0.9*r* to *r*, where *r* is set to 500 m. The path loss of large-scale fading η can be calculated depending on Equation (2). The sum rate of the system rate was employed as the performance metric, and the analysis of the rate actually achieved is shown in Equation (21). By repeating the Monte-Carlo simulation 1000 times, we compared the sum rate of JPFAS with three other conventional equal-power-allocated schemes, namely the perfect feedback scheme (PFS), equal feedback scheme (EFS), and dynamic feedback scheme (DFS) under different scenarios *K* = 3, *N* = 2, *D* = 1; *K* = 3, *N* = 4, *D* = 1 and *K* = 3, *N* = 4, *D* = 2. The power of Gaussian noise was given by −113 dB and the number of sum feedback bits $B_{f}$ was assumed to be 20.

**Figure 3:**
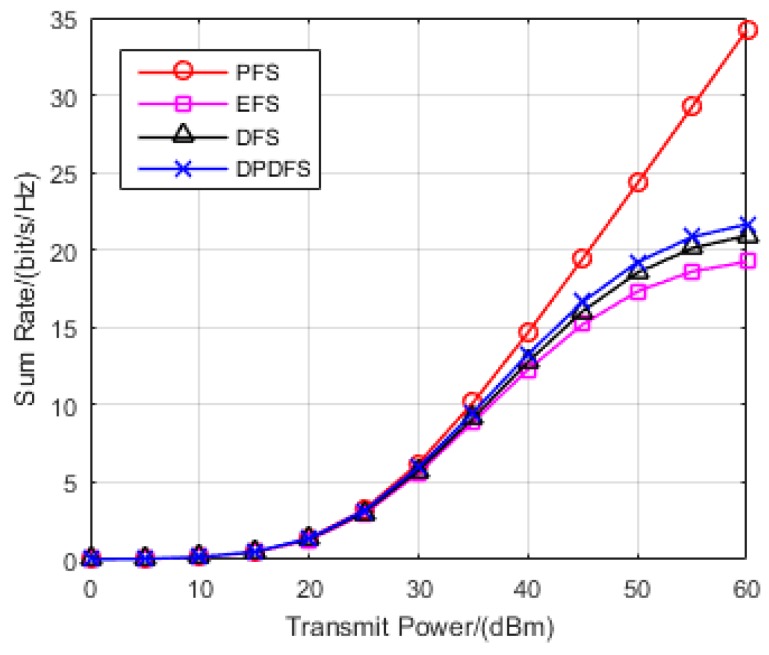
Comparison of the sum rates of PFS, EFS, DFS and JPFAS in the *K* = 3, *N* = 2, *D* = 1 symmetric networks.

First, we compared the sum rate of four schemes (PFS, EFS, DFS and JPFAS) under the scenario with *K* = 3, *N* = 2, *D* = 1. As is shown in Figure 3, when the transmitted power was lower than 35 dBm, the sum rate of the four schemes was equal due to the weak interference. When the transmitted power was larger than 35 dBm, it was expected that the sum rate of the three limited feedback schemes would be much lower than a perfect CSI feedback scheme, because the performance of the IA system was significantly degraded by the interference leakage. However, the joint power and feedback bit allocation interference alignment scheme we proposed was larger than the equal feedback scheme, and similar to the dynamic feedback scheme in terms of the sum rate because the residual interference was reduced by JPFAS.

**Figure 4:**
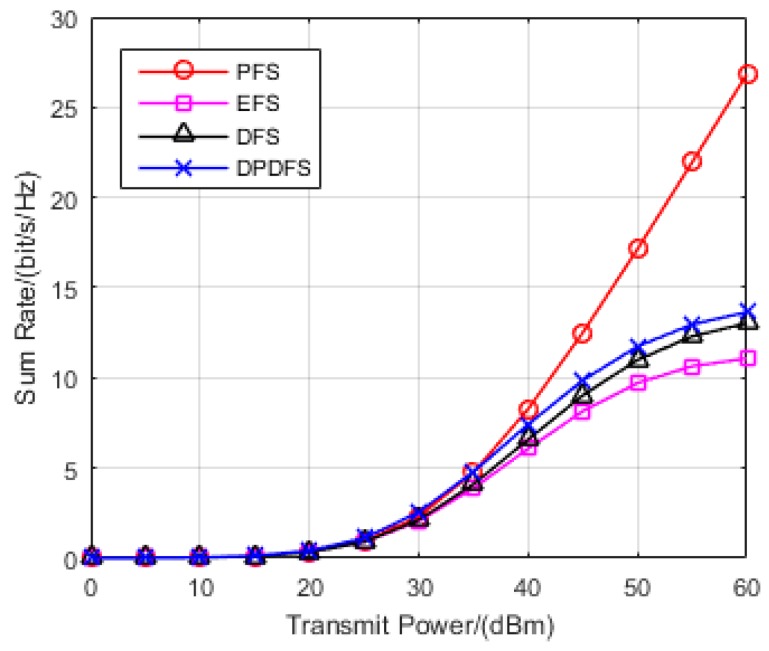
Comparison of the sum rates of PFS, EFS, DFS, and JPFAS in the *K* = 3, *N* = 4, *D* = 1 symmetric networks.

Secondly, we compared the sum rate of the four schemes (PFS, EFS, DFS and JPFAS) under the scenario with *K* = 3, *N* = 4, *D* = 1. It can be seen from Figure 4 that the total rate loss was small when the transmitted power of data streams was lower than 35 dBm. However, when the transmission power was larger than 35 dBm, as the transmitted power increases, the sum rate of the three limited feedback schemes becomes much lower than the perfect CSI feedback scheme, because the power of the interference streams scales with the transmitted power. It can clearly be seen that, the JPFAS we proposed outperformed the equal feedback bits allocation scheme, and can achieve comparable performance of the dynamic feedback scheme. This was because residual interference in this scenario consisted of only inter-user interference and none intra-user interference. JPFAS reduced the residual interference, increased the transmitted power and reduced the sum rate loss. Compared with Figure 3, JPFAS still achieved a better sum rate than other limited feedback schemes even though the quantization error was scaled with the number of antennas.

**Figure 5:**
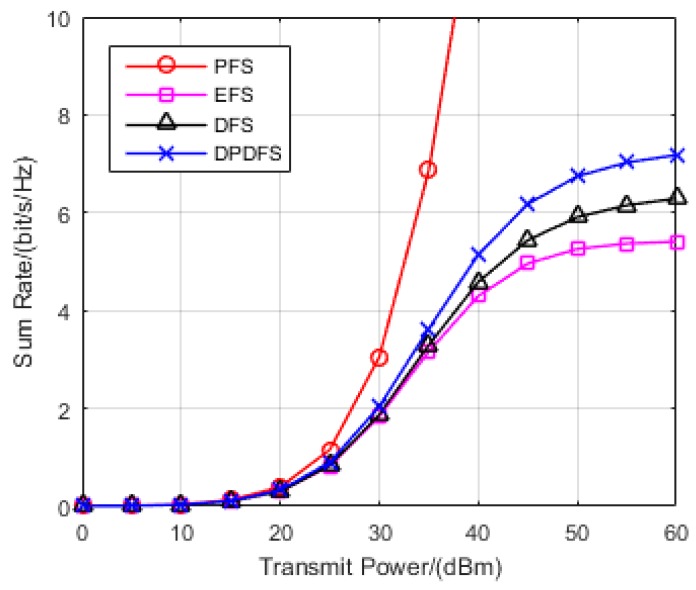
Comparison of the sum rate of PFS, EFS, DFS, and JPFAS in the *K* = 3, *N* = 4, *D* = 2 symmetric networks.

Furthermore, in order to validate the performance of the proposed scheme, we compared the sum rate of conventional feedback schemes and JPFAS in the scenario of multiple data streams. Considering *K* = 3, *N* = 4, *D* = 2, Figure 5 shows significant advantages of JPFAS over conventional schemes when the transmitted power was larger than 30 dBm. As intra-user interference was much greater than inter-user interference in this scenario, the sum rate of limited feedback schemes was much lower than perfect CSI feedback schemes, and the results are shown in Figure 5. Besides, by distributing the optimal power of multiple data streams and channel feedback bits, the proposed JPFAS still achieved better performance of the sum rate than the other two feedback schemes. Therefore, the JPFAS has an effect on reducing the residual interference and throughput loss.

## 6. Conclusions and Future Work

In this paper, a novel joint power and feedback-bit allocation scheme for IA has been proposed to mitigate the throughput loss caused by limited feedback that could not be reduced effectively by traditional IA schemes in wireless sensor networks. By exploiting the throughput of IA with limited feedback, the upper-bound of the rate loss was analyzed. Depending on the proposed scheme, the transmitted power of multiple data streams was allocated based on the communication link that the receiver desires. Moreover, the feedback bits were dynamically allocated on the basis of heterogeneous path loss. Considering the total transmitted power and heterogeneous path loss, the analytical sum of the average rate of the proposed JPFAS has been formulated in situations where different data streams were transmitted. The simulation results demonstrated that the proposed JPFAS outperforms conventional IA systems with limited feedback, and it achieved less residual interference and better performance in the system throughput.

In this paper, intra-user residual interference is much more than the residual interference of inter-users when the number of data streams is more than one. Therefore, it is important for each user to select the optimal number of data streams in terms of throughput. In our future work, we will investigate this problem, and develop a strategy for each user to select the optimal number of data streams to achieve even better performance.

## References

[1] Rafique Z., Seet B.-C., Al-Anbuky A. (2013). Performance analysis of cooperative virtual MIMO systems for wireless sensor networks. Sensors.

[2] Islam M.R., Han Y.S. (2011). Cooperative mimo communication at wireless sensor network: An error correcting code approach. Sensors.

[3] Yuan Y., He Z., Chen M. (2006). Virtual MIMO-based cross-layer design for wireless sensor networks. IEEE Trans. Vehic. Technol..

[4] Sung H., Park S.-H., Lee K.-J., Lee I. (2010). Linear precoder designs for K-user interference channels. IEEE Trans. Wirel. Commun..

[5] Cadambe V.R., Jafar S.A. (2009). Interference alignment and the degrees of freedom of wireless networks. IEEE Trans. Inf. Theory.

[6] Cadambe V.R., Jafar S.A. (2008). Interference alignment and degrees of freedom of the-user interference channel. IEEE Trans. Inf. Theory.

[7] Bresler G., Cartwright D., Tse D. (2014). Feasibility of interference alignment for the MIMO interference channel. IEEE Trans. Inf. Theory.

[8] Tang J., Lambotharan S. (2013). Interference alignment techniques for mimo multi-cell interfering broadcast channels. IEEE Trans. Commun..

[9] Ntranos V., Maddah-Ali M.A., Caire G. (2015). Cellular interference alignment. IEEE Trans. Inf. Theory.

[10] Kim K., Jeon S.-W., Kim D.K. (2015). The feasibility of interference alignment for full-duplex MIMO cellular networks. IEEE Commun. Lett..

[11] Huang L., Zhu G., Du X. (2013). Cognitive femtocell networks: An opportunistic spectrum access for future indoor wireless coverage. IEEE Wirel. Commun..

[12] Luo Y., Du H., Ratnarajah T., Wilcox D. Performance of homogeneous and asynchronous ad hoc network with interference alignment. Proceedings of the IEEE International Conference on Communications (ICC).

[13] Gomadam K., Cadambe V.R., Jafar S.A. (2011). A distributed numerical approach to interference alignment and applications to wireless interference networks. IEEE Trans. Inf. Theory.

[14] Peters S.W., Heath R.W. (2011). Cooperative algorithms for MIMO interference channels. IEEE Trans. Veh. Technol..

[15] Wu Z., Jiang L., Ren G., Zhao N., Zhao Y. (2015). A novel joint spatial-code clustered interference alignment scheme for large-scale wireless sensor networks. Sensors.

[16] Jiang L., Wu Z., Ren G., Wang G., Zhao N. (2015). A rapid convergent low complexity interference alignment algorithm for wireless sensor networks. Sensors.

[17] Love D.J., Heath R.W., Lau V.K., Gesbert D., Rao B.D., Andrews M. (2008). An overview of limited feedback in wireless communication systems. IEEE J. Sel. Areas Commun..

[18] Thukral J., Bolcskei H. Interference alignment with limited feedback. Proceedings of the IEEE International Symposium on Information Theory.

[19] Krishnamachari R.T., Varanasi M.K. (2013). Interference alignment under limited feedback for MIMO interference channels. IEEE Trans. Signal Process..

[20] Roh J.C., Rao B.D. (2006). Transmit beamforming in multiple-antenna systems with finite rate feedback: A vq-based approach. IEEE Trans. Inf. Theory.

[21] Santipach W., Honig M.L. (2009). Capacity of a multiple-antenna fading channel with a quantized precoding matrix. IEEE Trans. Inf. Theory.

[22] Lee H.-H., Ko Y.-C. Interference alignment with random vector quantization for MIMO interference channels. Proceedings of the VTC Fall 2012: IEEE Vehicular Technology Conference.

[23] Cho S., Huang K., Kim D., Seo H. (2012). Interference alignment for uplink cellular systems with limited feedback. IEEE Commun. Lett..

[24] Kim J.-S., Moon S.-H., Lee S.-R., Lee I. (2012). A new channel quantization strategy for MIMO interference alignment with limited feedback. IEEE Trans. Wirel. Commun..

[25] Farhadi H., Wang C., Skoglund M. On the throughput of wireless interference networks with limited feedback. Proceedings of the 2011 IEEE International Symposium on Information Theory (ISIT).

[26] Chen X., Yuen C. (2014). Performance analysis and optimization for interference alignment over MIMO interference channels with limited feedback. IEEE Trans. Signal Process..

[27] Chen X., Yuen C. (2016). On interference alignment with imperfect csi: Characterizations of outage probability, ergodic rate and ser. IEEE Trans. Veh. Technol..

[28] Anand K., Gunawan E., Guan Y.L. (2013). Beamformer design for the MIMO interference channels under limited channel feedback. IEEE Trans. Commun..

[29] Schreck J., Wunder G., Jung P. (2015). Robust iterative interference alignment for cellular networks with limited feedback. IEEE Trans. Wirel. Commun..

[30] Cho S., Chae H., Huang K., Kim D., Lau V.K., Seo H. Efficient feedback design for interference alignment in MIMO interference channel. Proceedings of the 2012 IEEE 75th Vehicular Technology Conference (VTC Spring).

[31] Rao X., Ruan L., Lau V.K. (2013). Limited feedback design for interference alignment on MIMO interference networks with heterogeneous path loss and spatial correlations. IEEE Trans. Signal Process..

[32] Zhang H., Jiang C., Beaulieu N.C., Chu X., Wang X., Quek T.Q. (2015). Resource allocation for cognitive small cell networks: A cooperative bargaining game theoretic approach. IEEE Trans. Wirel. Commun..

[33] Zhang H., Jiang C., Mao X., Chen H.-H. (2016). Interference-limited resource optimization in cognitive femtocells with fairness and imperfect spectrum sensing. IEEE Trans. Veh. Technol..

